# Development and validation of new figural scales for female body dissatisfaction assessment on two dimensions: thin-ideal and muscularity-ideal

**DOI:** 10.1186/s12889-020-09094-6

**Published:** 2020-07-16

**Authors:** Christina Ralph-Nearman, Ruth Filik

**Affiliations:** 1 School of Psychology, University of Nottingham, Nottingham, UK; 2 Presently at Laureate Institute for Brain Research, 6655 S Yale Avenue, Tulsa, OK 74133 USA

**Keywords:** Female body dissatisfaction, Thin-ideal, Eating disorders, Muscularity-ideal, Fit-ideal, Fitspiration, Body-image assessment, Body image, Drive for muscularity

## Abstract

**Background:**

Body dissatisfaction influences women’s mental and physical health. To date, most research has focused on body dissatisfaction in relation to the ‘thin-ideal’. Thus, the association between body dissatisfaction, eating disorder symptomatology and muscularity-ideal in women is less clear. Lack of understanding is underpinned by the lack of reliable and valid muscularity-related assessments for women. To address this need, we developed, tested and re-tested two new body dissatisfaction scales: The Female Body Scale (FBS; adiposity dimension) and Female Fit Body Scale (FFITBS; muscularity dimension).

**Methods:**

One hundred and fifty-two women in the United Kingdom rated which body figure best represented their current and ideal body, completed the Eating Disorder Examination Questionnaire (EDE-Q 6.0), and their body composition was measured. During re-test, the EDE-Q 6.0 and Drive for Muscularity Scale (DMS) were completed.

**Results:**

Both the FBS and the FFITBS were found to be valid and reliable, and distinct types of body dissatisfaction were identified. Higher EDE-Q scores corresponded with greater body dissatisfaction scores on both the FBS and FFITBS. Thin-ideal (FBS) and larger/muscularity-ideal (FFITBS) body dissatisfaction predicted higher scores on the DMS. The muscularity scale (FFITBS) uniquely revealed that 28% of participants indicated body dissatisfaction toward the *larger*-muscularity-ideal.

**Conclusions:**

Results reveal distinct dimensions of body dissatisfaction. These new, validated scales may be utilized to quickly identify eating disorder risk in women as a preventative assessment for clinicians and inform female-focused body-image and eating disorder research.

## Background

Body image disturbance has been strongly linked to eating disorder symptomatology and is reported to be a key factor in eating disorder relapse [18]. Most research on body image and body dissatisfaction in the past 20 years has primarily focused on females’ idealization of a thin body. The ‘thin-ideal’ (traditionally a desire for a thin, non-fat nor muscular body figure) has been identified as a key factor in body dissatisfaction, body image disturbance, and eating disorder pathology, and is related to extreme behaviors with adverse effects, such as: eating restraint, purging, and over-exercising [31]. As eating disorders directly take the lives of at least one person every 62 min [10], and body image disturbance is a key factor for these disorders, it is imperative to understand distinct dimensions of body dissatisfaction. In this current study, we will focus on muscularity-ideal, as well as thin-ideal, to get a more comprehensive picture of body dissatisfaction in women. Specifically, we will develop and test two new body dissatisfaction scales which will allow us to assess levels of body dissatisfaction relating to both adiposity- and muscularity-related concerns.

While most studies and assessments regarding female-related body dissatisfaction and eating disorder tendencies are designed to reveal body dissatisfaction toward the ‘thin-ideal’ on an emaciated to adipose dimension, growing evidence suggests that many women currently desire a body figure within a muscularity dimension. This dimension has an array of terminologies; both for the thin-ideal body figure which includes muscularity (e.g., ‘fit-ideal’, ‘toned-ideal’, ‘athletic-ideal’), which we will refer to as ‘thin-muscularity-ideal’, as well as the larger muscularity-ideal (desire for a larger, more muscular figure) [15]. For example, there is a recent emergence of women promoting the idealization of a muscular, ‘toned’, ‘fit’, or ‘athletic’ body ideal, rather than the ‘skinny’ body figure that is commonly seen within popular culture; both may be a risk-factor for eating disorder and body image-related disturbances [36]. The pursuit of a muscular ideal body figure may include physical and mental risks, such as: excessive or dangerous exercise practices, drug misuse, unbalanced and restrictive food intake, which result in compromised immunity, organ damage (e.g., kidney), and mental health problems (e.g., depression, mood swings, anxiety, etc.)(e.g., [19]). In 14 months (mid-October, 2018 to May, 2020), there was an increase of over 68.6 million posted pictures (from 76.2 million to 144.8 million) on the social media site Instagram under ‘#fitspiration’ and ‘#fitspo’ (the combination of fit + inspiration), making it apparent that there is a dramatic rise in women who shun ‘skinny’ and exalt ‘strong’. Women also have recently been shown to prefer an extremely thin and muscular body rather than merely an extremely thin body [2]. However, the thin-muscularity-ideal (thin-ideal with muscularity) may also be related to many of the behaviors associated with eating disorder symptomatology, such as: restricting food, purging, vomiting, over-exercising, and doing ‘whatever it takes’ to obtain an often unattainable ‘fit’, ‘strong’, ultra-toned body (e.g., [16]).

Despite the evidence that the number of women who idealize a body within the muscularity-ideal dimension (whether toward the thin-muscularity or larger-muscularity figure) is increasing, still, muscularity-ideal is most often associated with and researched in men, where it is reported to be related to the drive for muscularity [28], depression, reduced well-being, and overall dissatisfaction with life [6]. The relationship between body dissatisfaction, eating disorder symptomatology and muscularity-ideal in women is less clear. To date, there are few studies regarding the relationship between thin-muscularity-ideal (i.e., thin-ideal which includes muscularity body figure) and muscularity-ideal (i.e., larger/more muscular ideal body figure) in population samples of women with validated scales, and the results are disparate. For instance, in one study, muscularity-ideal was not related to body dissatisfaction [1], but in another study it was solely related to body dissatisfaction [37]. Both of these recent studies used linguistically-based self-report assessments, and found that the desire for a ‘fit-ideal’ (i.e., thin-muscular-ideal) body figure did not counteract the destructive behaviors related to the ‘thin-ideal’ (i.e., desire for a thinner body without muscle) body figure, such as bulimic symptoms, negative affect, and dieting.

The lack of understanding relating to the thin- or muscularity-ideal pertinent to women is also reflected in the lack of measurements available to assess body image. According to self-discrepancy theory, body dissatisfaction has been measured by the discrepancy between an individual’s perceived current body figure and their desired body figure, which has been reported to be highly related to eating disorder symptomatology and behaviors [3, 32], and a significant risk factor for other mental health conditions, such as depression [30].

Figural rating scales, in which an individual selects their perceived current and ideal body figure from an array of figures, have been shown to be a quick and rigorous method to both: 1) successfully classify individuals’ body size, be highly related to self-reported body mass index (BMI = kg/m^2^) in diverse and substantial populations, and 2) to robustly measure attitudinal and perceptual dimensions of body image distortions [4, 20, 27]. The majority of studies using visually-based scales have primarily focused solely on one-dimension (emaciated to obese) of body dissatisfaction for women, that is, the ‘thin-ideal’ body figure (i.e., ‘skinny’ with no muscle or fat), and do not include an independent measure of ‘muscularity-ideal’ (i.e., emaciated to muscularity dimension). The most frequently used figure scale to quickly and validly measure body dissatisfaction has been the nine-figured Stunkard Figure Rating Scale (SFRS [34]), which has been shown to possess comparable to, or higher validity than, a multitude of other figure scales comparing participants’ BMI to their dissatisfaction scores [11, 33]. However, there are limitations to using the SFRS, such as solely measuring a single dimension (emaciated to obese spectrum of body range). In addition, many other body figure rating scales that do incorporate muscularity have generally been found to not provide large or balanced enough body figures [7], exclude the neck and face area, which may involve important body-size concerns such as a double chin or chubby cheeks (e.g., [11, 26]), or are unrealistic/cartoon-like. Also, some papers reporting scale development do not provide any test-retest reliability results and only provide self-report BMI which is a subjective, perhaps unreliable marker [26]. Even one of the most commonly used current tools for measuring fat- and muscularity-related body dissatisfaction, the Somatomorphic Matrix [14] is reported not to have adequate test/retest reliability in women, and also only assesses fat/muscularity as a single dimension (e.g., [7]).

The lack of investigation surrounding muscularity-ideal in females has also resulted in few validated and reliable assessments which specifically assess muscularity concerns in female community samples. Most of these are language-based tools (e.g., [29, 37]). The most widely used tool, the Drive for Muscularity Scale (DMS [22]) is also a language-based assessment for the preoccupation with growing muscularity, showing evidence of validity and reliability in different cultural contexts (e.g., [9, 13]). In any case, it is important to consider that language-based assessments may not be suitable for all individuals, for example, those with poor literacy. In addition, visually-based stimuli may be more sensitive in identifying body dissatisfaction and eating disorders than word-based stimuli [38]. Thus, there is a need for new reliable and validated female-specific body dissatisfaction measures which independently assess the two distinct dimensions, including muscularity dissatisfaction as well as the thin-ideal, and do not heavily rely on language.

In response to this need, we have developed the new *Female Body Scale* (FBS) (depicting a series of nine female bodies ranging from emaciated to obese) and *Female Fit Body Scale* (FFITBS) (depicting a series of nine female bodies ranging from emaciated to very muscular). These new visually-based assessments also provide standardized measures for two contrasting assessments of body dissatisfaction in women. This will allow us to identify whether the majority of women’s body dissatisfaction is focused toward the thin/emaciated-ideal, larger/adipose-ideal, thin/muscularity-ideal, or larger/muscular-ideal trend, and how these different types of body dissatisfaction in women are related to eating disorder symptomatology and the drive for muscularity.

Thus, the main aim of the current study is to develop, test and re-test these two new female body dissatisfaction scales. These scales were also compared to one of the most widely used body dissatisfaction scales for over 30 years (Stunkard Figure Rating Scale; SFRS), and the published test/retest reliability results [5] for one of the most commonly used current body dissatisfaction scales (Somatomorphic Matrix [14]), which includes both muscularity and adiposity for females.

Overall, we aim to assess whether the new scales are valid, reliable, and subjectively representative of women’s current and ideal body figure, from which we will calculate their level of body dissatisfaction. In addition, we wish to assess construct validity in terms of whether the ratings given on each scale correspond to women’s measured body measurements (i.e., their body mass index, fat-, and muscularity-percentage), their eating disorder symptomatology (as assessed by the Eating Disorder Examination Questionnaire; EDE-Q [12]), and provide a visual assessment which may quickly elucidate how specific aspects of overall body dissatisfaction in women may predict their drive for muscularity (as measured by the DMS [22]).

## Methods

### Participants

This study was approved by the appropriate ethical review board. A local community sample of 152 native English-speaking women participated for a small inconvenience allowance or course credit. Ages ranged from 18 to 59 years (*M* = 21.78, *SD* = 5.82). Measured Body Mass Index (BMI = Kg/ M^2^) ranged from 15.60 (Underweight) to 39.20 (Obese) (*M* = 23.04, *SD* = 4.20), Body Fat Percentage (BFP) ranged from 9.20% (Essential Fat) to 49.80% (Obese) (*M* = 23.22%, *SD* = 7.19), and measured Body Muscle Percentage (BMP) ranged from 32.80% to 50.8% (*M* = 38.84%, *SD* = 2.88).

### Scale development

**Fig. 1:**
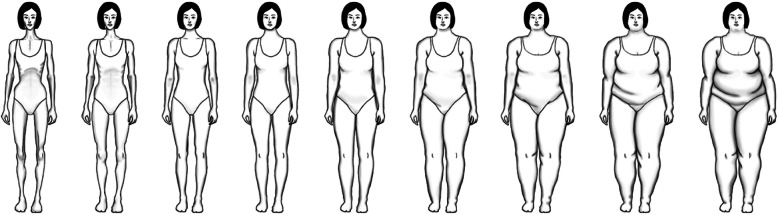
Female Body Scale

**Fig. 2:**
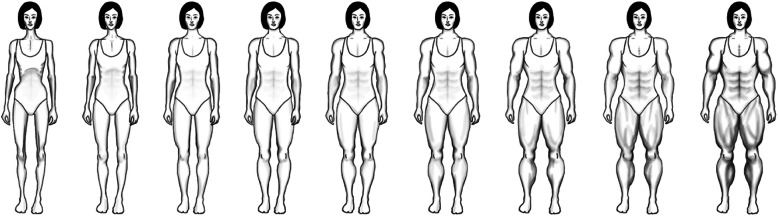
Female Fit Body Scale

We developed the FBS and the FFITBS from actual female body figures, with help from a professional artist/graphic designer. Firstly, they modeled the thinnest (emaciated), and largest (obese and muscular) figures (Fig. 1 and 9 in each scale) from photographs of anorexic, obese, and muscle-lifting females. They then modeled each graduating body figure size to photographs of actual women, precisely drawing and contouring each increasing figure 10% in width between each body figure. Finally, the figures were scanned into a computer, and Adobe Photoshop was used to verify the uniform increase between figures from most emaciated to largest figure in each scale, using transparency layers to ensure only the body width and not height were increased 10% between figures. By following this procedure, the figures accurately represented systematically increasing sizes of actual female body figures across the nine different figures in each scale. This provided study participants with the option to select their current, and then ideal body figure, both in relation to their level of body fat (FBS) and their level of fitness/muscularity (FFITBS) (see Figs. 1 and 2).

### Procedure

After signing an informed consent, participants were presented with three body scales (the FBS and FFITBS along with the SFRS, for comparison) using Qualtrics software. They were asked to indicate which of the nine body figures on each scale best represented their current figure, and then, with the same scale but on a new screen, asked to indicate their ideal body figure. After this, they were asked to indicate 1) which of the three scales (i.e., FBS, FFITBS, or SFRS) best represented how they currently look, 2) which scale (overall) best represented how they would ideally like to look, and 3) which scale (overall) best represented how others see them. Finally, they filled out the EDE-Q, and then, bioelectrical impedance analysis was used to measure and calculate their actual BMI, BFP and BMP. This procedure was followed by a thorough debrief, including clinical resources.

**Fig. 3:**
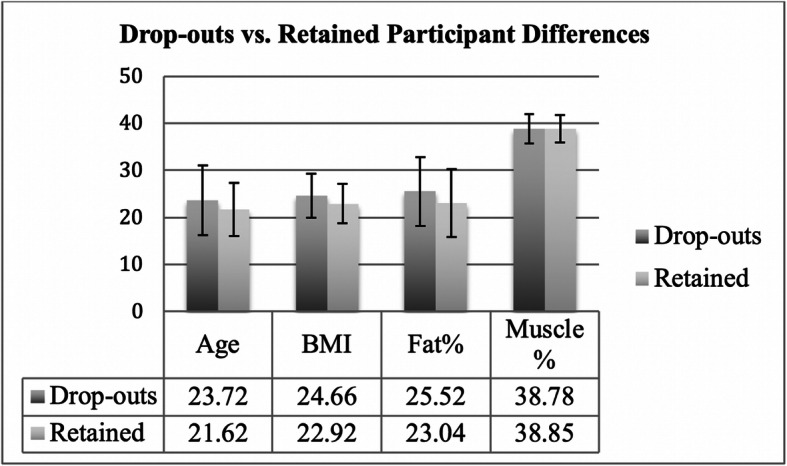
Age, Body Mass Index (BMI), Body Fat Percentage, and Body Muscularity Percentage differences between drop-outs and retained participants. Error bars represent SD.

The Time 1 test (initial lab test) was followed by a re-test and manipulation check in Time 2, 1 to 3 weeks (*M* = 1.42, *SD* = 0.46) after Time 1, in which all of the participants were emailed a link and original code to repeat the initial portion of the experiment, as well as the DMS. Of the 152 original participants, 141 also participated in Time 2, representing a response rate of 92.76%. These participants ranged from 18 to 59 years old (*M* = 21.58, *SD* = 5.60), BMI ranged from 15.60 to 39.20 (*M* = 22.92, *SD* = 4.15), BFP ranged from 9.20% (Essential Fat) to 49.8% (Obese; *M* = 23.04%, *SD* = 7.17), and BMP ranged from 32.80% to 50.80% (*M* = 38.85%, *SD* = 2.87). Independent samples t-tests confirmed that there were no significant differences in terms of age, BMI, BFP, or BMP between Time 1 and Time 2 participants (*t*s < 1.32, *ps* > .19) (see Fig. 3 BMI, BFP, BMP Test and Re-test Differences).

As a manipulation check in Time 2, a computer-generated randomization was applied to the order of the nine body figures on each of the two new body scales, and participants were instructed to click and drag each of the figures up or down within the program to re-arrange them from thinnest to largest body figure in each separate scale (FBS and FFITBS).

### Other scales and measurements

#### Actual body measurements

Participants were first asked to remove any heavy outer clothing layers, shoes, and socks. Height was measured using a stadiometer, and a bioelectrical impedance analyzer was used to measure each participant’s actual fat- and muscularity-percentage. Then, each participant’s BMI (Kg/M^2^) was computed.

### Eating disorder symptomatology

The EDE-Q 6.0 [12] is the only measure of a person’s level of eating disorder pathology and monitoring of eating disorder progression recommended in England [24]. Global eating disorder scores are made up of 23 items assessing eating disorder related symptomatology from the previous 28 days from 0 (Not at all) to 6 (Markedly), on a 7-point scale, thus meeting the ≥5 category rule to be considered as an “ordinal approximation of a continuous variable” [17, 25, 35, 39]. Global scores are the average of the scores on four subscales assessing; eating restraint, eating concern, body shape concern, and weight concern. The EDE-Q 6.0 scale was reliable as Cronbach’s alpha (*N* = 152) was .95, 95% CI[.93, .96] (Global); .79, 95% CI[.68,.81](Restraint); .94, 95% CI[.83,.91](Shape); .77, 95% CI[.65,.79](Eating); and .83, 95% CI[.72,.84](Weight). Participants’ scores ranged from .00 (no eating disorder symptomatology) to 5.0 (over the clinical eating disorder threshold) (e.g., [8, 23]) out of 6.0 (see Table 1).

**Table 1:** EDE-Q 6.0 Scores

Facet	Actual Range	Possible Range	Mean	SD
EDE-Q Global	0 to 5	0 to 6	1.91	1.22
WC Total	0 to 6	0 to 6	2.42	1.49
EC Total	0 to 5	0 to 6	1.72	1.31
SC Total	0 to 6	0 to 6	2.83	1.53
RC Total	0 to 6	0 to 6	1.38	1.30

*WC* weight concern, *EC* eating concern, *SC* shape concern, *RC* restraint concern

### Drive for muscularity scale

DMS [22] is a valid and reliable 15-item scale with both muscularity behaviors (i.e., *I lift weights to build up muscle.*) and muscularity desire (i.e., *I think I would feel more confident if I had more muscle mass.*) rated from 1–*Always* to 6–*Never* (reverse scored) to determine drive for muscularity level. All 15 items load onto a single global “drive for muscularity” facet for women [21]. Participants’ DMS scores ranged from 15 (low drive for muscularity) to 68 (high drive for muscularity; *M* = 29.64; *SD* = 11.35), and the scale was reliable as Cronbach’s Alpha was .93 (*N* = 141), 95% CI[.87, .92].

### Analysis

In both Time 1 and Time 2, the range and mean for each of the three body scales (FBS, FFITBS, and SFRS) were calculated, as was the test-retest reliability over a one-to-three-week period. The percentage of correctly ordered body sizes in each scale (from 1 (emaciated figure) to 9 (obese figure); and 1 (emaciated figure) to 9 (largest muscular figure)) for the manipulation check in Time 2 was also calculated.

Construct validity was then determined in three ways. Firstly, we examined the degree of correspondence between participants’ actual body measurements (i.e., BMI, BFP, and BMP), and their current body rating in each of the three scales. Secondly, construct validity pertaining to the desire for muscularity was measured by the degree of correspondence between each scale’s body dissatisfaction ratings (calculated in terms of participants’ ideal body figure choice minus their current body figure choice in each scale) and scores on the DMS. Thirdly, we examined the degree of correspondence between each participant’s current and ideal body figure choice and body dissatisfaction scores (current scores minus ideal scores) on each scale and their eating disorder symptomatology (EDE-Q 6.0 scores).

## Results

### Manipulation check

Results from the manipulation check showed that the order of the increase of body size in each scale was consistent for each of the new scales, with 96.93% and 94.48% of participants accurately ordering each one of the nine body figures sequentially in the FBS and FFITBS, respectively.

### Current figure choices, ideal figure choices, and body dissatisfaction scores

Approximately 70% of participants in both Time 1 (*N* = 152) and Time 2 (*N* = 141) chose a thinner ideal (less adiposity) body figure with greater body dissatisfaction displayed on both the SFRS and FBS (measuring from emaciated to obese). In comparison, less than 50% chose a thinner (less muscular) ideal body figure on the muscularity-dimension on the FFITBS (see Table 2 Descriptive Statistics for Time 1 Self-rating Selections, and Table 3 Descriptive Statistics for Time 2 Self-rating Selections).

**Table 2:** Descriptive Statistics for Time 1 Self-rating Selections (N = 152)

Scale	Figure Choice	Actual Range		Possible Range	Mean		*SD*
SFRS	Current	1 to 8		1 to 9	3.86		1.20
SFRS	Ideal	1 to 5		1 to 9	2.82		.74
SFRS	BD Scores	-4 to 2		-8 to 8	−1.05		1.07
FBS	Current	2 to 9		1 to 9	4.27		1.25
FBS	Ideal	1 to 5		1 to 9	3.22		.80
FBS	BD Scores	−4 to 3		−8 to 8	−1.05		1.11
FFITBS	Current	2 to 7		1 to 9	4.01		1.00
FFITBS	Ideal	1 to 7		1 to 9	3.71		1.11
FFITBS	BD Scores	−3 to 3		−8 to 8	−.30		1.22

*BD Scores* body dissatisfaction scores (ideal figure minus current figure), *SFRS* Stunkard Figure Rating Scale, *FBS* female body scale, *FFITBS* female fit body scale

**Table 3:** Descriptive Statistics for Time 2 Self-rating Selections (N = 141)

Scale	Figure Choice	Actual Range	Possible Range	Mean		*SD*
SFRS	Current	1 to 8	1 to 9	3.80		1.33
SFRS	Ideal	1 to 7	1 to 9	2.85		.83
SFRS	BD Scores	−5 to 2	−8 to 8	−.95		1.19
FBS	Current	2 to 9	1 to 9	4.35		1.30
FBS	Ideal	2 to 5	1 to 9	3.25		.78
FBS	BD Scores	−5 to 3	−8 to 8	−1.10		1.19
FFITBS	Current	1 to 7	1 to 9	3.87		1.04
FFITBS	Ideal	2 to 8	1 to 9	3.67		1.07
FFITBS	BD Scores	−4 to 4	−8 to 8	−.19		1.24

*BD Scores* body dissatisfaction scores (ideal figure minus current figure), *SFRS* Stunkard Figure Rating Scale, *FBS* female body scale, *FFITBS* female fit body scale

**Fig. 4:**
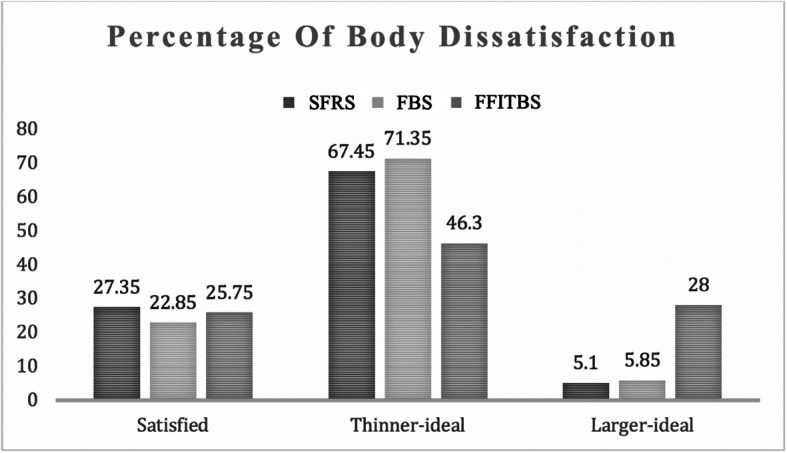
Percentage of Body Dissatisfaction. SFRS = Stunkard Figure Rating Scale; FBS = Female Body Scale; FFITBS = Female Fit Body Scale

On all three scales participants mainly rated themselves as body dissatisfied toward the thin-ideal rather than satisfied or toward the larger-ideal. However, 28% of participants reported that they were body dissatisfied, desiring a larger, more muscular body on the FFITBS, compared with < 6% on the SFRS and FBS (Fig. 4).

### Which scale best represents current, others’ view, and ideal body sizes overall?

Participants consistently reported which scale best represented: their current body, how others view them, and their ideal body size overall. These were consistent between Time 1 and Time 2 (*t*s < .67, *p*s > .51). That is, participants reported the FBS to best represent their perceived overall *current* body figure (67.1%; 66.0%), and that the FFITBS best represented their overall *ideal* body figure (64.5%; 65.2%), rather than the FBS (32.2%; 33.3%) or the SFRS (3.3, 1.4%). On average between Time 1 and Time 2 only 6.8% of participants reported viewing themselves as appearing most like the less-defined SFRS, but significantly more (11.7% on average) believed that *others* perceive their body in this way *t*(26) = 21.46, *p* < .001. These results point to consistency in selection of overall most representative scale for both the current and the ideal female body type, with the FBS and FFITBS being the two preferred scales to represent their current and ideal body type.

### Construct validity in relation to actual body measures

It is important to test the correspondence between the participants’ perceived current body size that they indicated by choosing one of the nine figures on the three scales, and their actual size as measured. Results showed that there was a strong correlation between an individual’s actual body mass index (BMI), body fat percentage (BFP), body muscle percentage (BMP), and current body size self-ratings for the total sample at both Time 1 and Time 2, on the SFRS, FBS, and FFITBS (see Table 4 Time 1 and 2 Correlations Between Current FBS Choice and Measures). Specifically, participants with lower BMI, lower body fat percentage, and higher body muscle percentage chose thinner perceived current body figures than participants with higher BMI, higher body fat percentage, and lower body muscle on all scales.

**Table 4:** *Time 1 and 2 Correlations Between Current Figure Body Scale Choice and Measures*

Body Scale	TIME 1			TIME 2		
	BMI *N* = 152	BFP *N* = 152	BMP *N* = 152	BMI *N* = 141	BFP *N* = 141	BMP *N* = 141
SFRS Current Figure	*rs* = .78**	*rs* = .79**	*rs* = −.62**	*rs* = .78**	*rs* = .79**	*rs* = −.62**
FBS Current Figure	*rs* = .76**	*rs* = .78**	*rs* = −.60**	*rs* = .76**	*rs* = .78**	*rs* = −.59**
FFITBS Current Figure	*rs* = .54**	*rs* = .57**	*rs* = −.44**	*rs* = .52**	*rs* = .55**	*rs* = −.43**

** = *p* < .001, *BMI* body mass index, *BFP* body fat percentage, *BMP* body muscularity percentage, *SFRS* Stunkard Figure Rating Scale, *FBS* female body scale, *FFITBS* female fit body scale

### Construct validity in relation to drive for muscularity

Also, construct validity was examined by the degree of correspondence between the DMS and body dissatisfaction scores (ideal figure minus current figure) obtained on each of the three scales. Results from these correlations confirmed a significant relationship between an individual’s DMS scores and body dissatisfaction (*rs* = .27, *p* = .001, *N* = 141) in a positive direction for the FFITBS. In contrast, individuals’ DMS scores were not significantly related to the dissatisfaction scores of either of the two scales measuring adiposity rather than muscularity/fitness: the SFRS, or the FBS, (*rs*s < .07, *p*s > .40, *N* = 141). Therefore, these results suggest that only the FFITBS body dissatisfaction scores may accurately identify women’s body dissatisfaction related to the drive for more muscularity.

Next, enter multi-linear regressions with the three scales’ body dissatisfaction scores as the predictor variables, and the DMS as the dependent variable, revealed that only the FFITBS greater body dissatisfaction scores, in the larger muscular-ideal direction, significantly predicted greater levels of drive for muscularity, as shown by the DMS scores (*b* = .35, *t*(137) = 3.84, *sr*^*2*^ = .31, *p* < .001). Additionally, greater FBS body dissatisfaction scores, in the thin-ideal direction, also significantly predicted greater levels of drive for muscularity, as shown by the DMS scores (*b* = −.34, *t*(137) = − 2.48, *sr*^*2*^ = −.20 *p* = .014). SFRS body dissatisfaction scores were not associated with the DMS scores (*b* = .16, *t* (137) = 1.21, *sr*^*2*^ = .10, *p* = .23). Tests indicated that multicollinearity was not a concern (Variance inflation factors ≤2.89).

### Construct validity in relation to the EDE-Q 6.0

Lastly, construct validity was examined by the degree of correspondence between the eating disorder symptomatology level (EDE-Q [12]) and body dissatisfaction scores. All three dissatisfaction scores significantly correlated (negatively) with EDE-Q global scores, as well as with facets of Restraint (e.g., “a definite desire to have an empty stomach with the aim of influencing their shape or weight”), Shape Concern (e.g., “desire to have a totally flat stomach”), and Weight Concern (e.g., “fear of gaining/maintaining weight”) in Time 1 and Time 2. Therefore, the idealization of a thinner body, whether with or without muscle, was associated with more eating disorder symptomatology for both the adiposity and muscularity dimensions. Eating Concern (e.g., “guilt, fear, and preoccupation with eating and calories”) was associated with women’s desire to be ‘skinny’ within the adiposity dimension, but not within the muscularity dimension in Time 1 or 2 (*rs* < −.15, *p* > .07), and only Weight Concern met the conservative Bonferroni correction (*p* < .008) in Time 2 for the FFITBS (see Table 5). So whilst muscularity dissatisfaction (FFITBS) was especially associated with Weight Concern, the thin-ideal within the adiposity dimension (FBS) was most strongly and consistently related to all facets of eating disorder symptoms.

**Table 5:** Time 1 and 2 Correlations Between Body Dissatisfaction Scores on Scales and EDE-Q Scores

TIME 1 *N* = 152				TIME 2 *N* = 141		
EDE-Q Facet	SFRS	FBS	FFITBS	SFRS	FBS	FFITBS
Global	*rs* = −.58**	*rs* = −.57**	*rs* = −.29**	*rs* = −.56**	*rs* = −.60**	*rs* = −.21, *p* = .012
Restraint	*rs* = −.42**	*rs* = −.41**	*rs* = −.25**	*rs* = −.38**	*rs* = −.43**	*rs* = −.19, *p* = .025
Shape Concern	*rs* = −.59**	*rs* = −.58**	*rs* = −.29*	*rs* = −.58**	*rs* = −.61**	*rs* = −.20, *p* = .020
Eating Concern	*rs* = −.37**	*rs* = −.34**	*rs* = −.15, *p* = .07	*rs* = −.39**	*rs* = −.46**	*rs* = −.10, *p* = .24
Weight Concern	*rs* = −.61**	*rs* = −.59**	*rs* = −.29**	*rs* = −.62**	*rs* = −.62**	*rs* = −.26*

** = *p* < .001; * = *p* = .002; *SFRS* Stunkard Figure Rating Scale, *FBS* female body scale, *FFITBS* female fit body scale

### Further test-retest reliabilities

Test-retest reliabilities for each scale were further confirmed by examining the correlations between current and ideal body size ratings, and body dissatisfaction scores between Time 1 and Time 2 (1 to 3 weeks later). Importantly, all correlations were significant for current body size, ideal body size, and body dissatisfaction scores between the Time 1 and Time 2 for the FBS, the FFITBS, and the SFRS (see Table 6).

**Table 6:** Test-Retest Correlations Between FFITBS, FBS, Female Somatomorphic Matrix [5], and Stunkard Figure Rating Scale

**Muscularity**	**FFITBS (*N* = 141)** **1–3 weeks**		**SM (*N* = 32)**^**+**^ **7–10 days**	
Current	*rs* = .75*		*r* = .54^+^	
Ideal	*rs* = .55*		*r* = .57^+^	
Dissatisfaction	*rs* = .63*		*r* = .35^+^	
**Adiposity**	**FBS(*N*** **= 141)** **1–3 weeks**		**SM(*N*** **= 32)**^**+**^ **7–10 days**	**SFRS(*N*** **= 141)** **1–3 weeks**
Current	*rs* = .86*	.	*r* = .75^+^	*rs* = .86*
Ideal	*rs* = .73*		*r* = .39^+^	*rs* = .68*
Dissatisfaction	*rs* = .78*		*r* = .56^+^	*rs* = .80*

*FFITBS* female fit body scale, *FBS* female body scale, *SM* somatomorphic matrix, *SFRS* Stunkard Figure Rating Scale; *p < .001; **+** = the *p*-values for [5] data are not known, and it is unclear if any of the 32 females dropped out at the retest in Time 2

The test/retest reliability results were compared with the published test/retest results of the most utilized current body scale for females that includes muscularity (Somatomorphic Matrix, [5]; see Table 6). Taken together, the results suggest that both new scales; the FFITBS and the FBS, are reliable, and exceed the reliability of the Somatomorphic Matrix body dissatisfaction scores.

## Discussion

Overall, as expected, women desired a thinner body compared to their perceived current body in both the adiposity and muscularity dimensions, but less so if the scale offered fit/muscular body figure choices. The FFITBS revealed that 28% of the participants expressed that they would like a larger, but more muscular-ideal body figure than their perceived current figure, rather than a more ‘fat’/ ‘obese’, or more ‘skinny’/'thin-ideal' figure. This suggests that women may often desire a lean, fit body, rather than merely a ‘skinny’ body if presented with a muscularity assessment option (see Fig. 4). These results also indicate that the new FFITBS detects body dissatisfaction in the direction of larger/more muscularity, which cannot be measured with the SFRS.

Results suggest the majority of our participants would like to be thinner than their perceived current body size, avoiding adiposity, and find a larger body more desirable if it is larger/muscularity-wise rather than larger/adiposity-wise figure. Also, muscularity-ideal was related to body dissatisfaction, which is in contrast to Bell et al. [1] who proposed that muscularity-ideal was not related to body dissatisfaction, but provides some support of Uhlmann et al. [37] who also reported this finding. However, importantly, unlike Uhlmann et al. [37] who also proposed that the desire for more muscularity is a protective factor from eating disorder symptomatology, we found that muscularity-related body dissatisfaction was also significantly related to aspects of eating disorder symptomatology, especially associated with concerns surrounding weight, and the drive for muscularity.

While muscularity-related body dissatisfaction on the FFITBS was significantly associated with drive for muscularity (DMS), the moderate correlation, indicates that these are perhaps overlapping but not identical concepts, as suggested in a male-related study [2]. It may be that some individuals may possess muscularity body dissatisfaction and concerns (shown on the FFITBS), but are not driven toward behaviors to change the muscularity of their body (shown on the DMS). Overall, body dissatisfaction toward a thinner-ideal *without* a muscularity dimension was found to be more strongly associated with, and encompassed more aspects of, overall eating disorder symptomatology risk, including restriction of food, and body shape, weight, and eating concerns, while thin-ideal (with or without desire for body muscle) may be more strongly indicative of weight concern-related eating disorder symptomatology and drive for muscularity in women.

The new FBS and FFITBS were shown to be valid and reliable, as well as being scales that our participants indicated best represent their current and ideal body figure. Each of these scales measures different dimensions of female body ideals and female body dissatisfaction and their association with the drive for muscularity and distinct eating disorder symptoms. The FBS and FFITBS also provide proportionally systematically increasing figure scales to measure body dissatisfaction. The new scales avoid limitations in past scales, such as reliance upon adequate literacy (for language-based questionnaires), and avoidance of; unrealistic, cartoonish, headless, unbalanced, disproportionate, or time-consuming measures of body dissatisfaction. Finally, these new scales provide reliable measures of distinct dimensions of body dissatisfaction in women, which may be utilized together or individually. As participants reported that the FBS best represented their overall *current* body figure, and that the FFITBS best represented their overall *ideal* body figure, in addition to these new scales which measure distinct aspects of body dissatisfaction, it may be of interest to develop a third scale combining features of FBS and FFITBS in a future study.

Limitations of this study include the need for culturally diverse versions of these scales, which we are in the process of developing. As the present study investigated a relatively small community sample, testing is currently underway with a broad range of samples, including international samples, clinical samples, and individuals outside ‘typical’ body size dimensions and/or perception (e.g., obesity, anorexia nervosa, muscle dysmorphia, fashion models, etc.). Also, although the test-retest correlations exceeded or were comparable to the most utilized figure rating scales for adiposity or muscularity (i.e., SFRS and somatomorphic matrix), these effects were moderate. This may be because body image perception and dissatisfaction changes quite often, and there is still a need to clarify the optimum timeframe between test and retest and expected reliability for visual scales. These effects may be somewhat influenced by the broader age range of our participants (18 to 59 years (*M* = 21.78, *SD* = 5.82)), which may have included a few women in menopause with differing body ideals. Investigating menopausal women with these tools may be a promising future study. Nevertheless, these primary results point to the importance of investigating different and distinct aspects of female body dissatisfaction and eating disorder symptomatology. As the ‘thin-ideal’ body dissatisfaction (with and without the dimension of muscularity) in participants was shown to be highly associated with detrimental eating disorder symptomatology, focusing on the distinct dimensions of female body dissatisfaction is an under-examined line of investigation which is crucially important. In addition, although body dissatisfaction toward the muscularity-ideal body in females in the general population is rarely investigated, or has not been apparent, this study reveals that a substantial percentage of women in the community population are body dissatisfied toward a larger/muscular-ideal, which was only detected on the FFITBS, and predicted greater drive for muscularity. Practical implications may include general practitioners utilizing these tools to quickly identify females who may be at-risk of eating disorder symptoms and drive for muscularity within the general population, as a preventative measure.

## Conclusions

In sum, two scales for females were developed, tested, and re-tested to quickly and robustly visually assess independent dimensions of muscularity-ideal, thin-muscularity-ideal, and the thin-ideal (emaciated to obesity), related to eating disorder symptomatology and drive for muscularity. Results show that both the FBS and FFITBS are valid and reliable visual body dissatisfaction measures, with the FBS detecting distinct aspects of adiposity-related body dissatisfaction, and FFITBS revealing muscularity-related body dissatisfaction, related to specific aspects of eating disorder symptomatology and drive for muscularity in women. These scales corresponded to women’s measured body composition (i.e. BMI, fat, and muscularity), and may be utilized jointly or independently to quickly and easily measure distinct dimensions of body dissatisfaction to detect the drive for thinness or muscularity, as well as aspects of eating disorder symptomatology in women. Testing is currently underway utilizing FFITBS and FBS in populations with clinically diagnosed psychiatric conditions, such as eating disorders and body dysmorphic disorder, as well as individuals outside the 'average' body size, such as obese individuals and fashion models.  

## Data Availability

We do not have permission to share data. Materials are available upon request of the corresponding author.
